# Combined supplementation of *Lactobacillus fermentum* and *Pediococcus acidilactici* promoted growth performance, alleviated inflammation, and modulated intestinal microbiota in weaned pigs

**DOI:** 10.1186/s12917-019-1991-9

**Published:** 2019-07-10

**Authors:** Shilan Wang, Bingqian Yao, Hang Gao, Jianjun Zang, Shiyu Tao, Shuai Zhang, Shimeng Huang, Beibei He, Junjun Wang

**Affiliations:** State Key Laboratory of Animal Nutrition, College of Animal Science and Technology, China Agricultural University, Beijing, 100193 China

**Keywords:** Weaned pigs, Growth performance, Inflammation, Intestinal microbiota, Combined *Lactobacillus fermentum* and *Pediococcus acidilactici*

## Abstract

**Background:**

Probiotics are important for pigs to enhance health and intestinal development, which are potential alternative to antibiotics. Many studies have reported the functions of single bacterial strain as probiotic on the animals. In this study, we evaluated effects of combined probiotics on growth performance, inflammation and intestinal microbiota in weaned pigs. One hundred and eight pigs, weaned at 28 day old (7.12 ± 0.08 kg), were randomly divided into the 3 dietary treatments with 6 pens and 6 pigs per pen (half male and half female). The experimental period lasted for 28 days and treatments were as follows: i. Control: basal diet; ii. Antibiotic: the basal diet plus 75 mg· kg^− 1^ chlortetracycline; and iii. Probiotics: basal diet plus 4% compound probiotics.

**Results:**

Supplementation probiotics improved average daily gain over the entire 28 days (*P* < 0.01) and feed efficiency in the last 14 days (*P* < 0.05) compared with the other two groups. Both probiotics and antibiotic supplementation decreased concentrations of serum pro-inflammatory cytokines interleukin-6 (*P* < 0.05) and interferon-γ (*P* < 0.01). Probiotics group had greater abundance of *Lactobacillus* in the caecal digesta and Firmicutes in the colonic digesta, while both probiotics and antibiotic supplementation inhibited *Treponema_2* and *Anaerovibrio* in the caecal digesta. Caecal acetic and propionic acid (*P* < 0.05) of probiotics group were higher than the other two groups, whereas concentrations of colonic lactic acid and propionic acid (*P* < 0.05) of antibiotic group were lower than control and probiotics groups.

**Conclusions:**

These findings suggest that combined supplementation of *Lactobacillus fermentum* and *Pediococcus acidilactici* regulate the gut health and improve the host ADG and F/G by decreasing serum pro-inflammatory factors (IL-6, IFN-γ), promoting beneficial bacteria (*Lactobacillus* in the caecal digesta and Firmicutes in the colonic digesta), enhancing production of short chain fatty acids, and inhibiting pathogens (*Treponema_2, Anaerovibrio* in the caecal digesta).

**Electronic supplementary material:**

The online version of this article (10.1186/s12917-019-1991-9) contains supplementary material, which is available to authorized users.

## Background

Weaning is accompanied by changes of intestinal microbiota composition, diarrhea and growth inhibition in weaned pigs [1, 2]. Supplementation of feed with antibiotics can promote growth and stabilize intestinal microbiota [3], but now antibiotics have to be limited in pig production due to increased resistance of microorganism to antibiotics. The intestinal microbiota plays a pivotal role in benefiting the health of the host animals [4]. Probiotics can stimulate the immune system of piglets, inhibit growth of pathogens, and modulate composition and activity of the original microbiota [4], which are potential alternative to antibiotic, especially for preventive effect.

Recent previous research has reported the functions of lactic acid bacteria strains as probiotics in animals [4]. Some *Lactobacillus* species can change host intestinal microbiota through producing lactic acid and other microbial compounds, and they may prevent colonization of pathogens via competitive exclusion [5, 6]. *Lactobacillus* could use nutrients that the host cannot metabolize, and thus affect physiological functions of animals, such as the general health and growth [7].

*Lactobacillus fermentum (L. fermentum)* strains improved the anti-oxidative defense system of weanling pigs [8] and prevented intestinal infections caused by enterotoxigenic *Escherichia coil* [9]. A previous study showed that *Lactobacillus fermentum* I5007 relieved the weaning stress by decreasing expression of proteins participated in stress response and increasing levels of proteins in relation to protein synthesis, and immune response [10]. Feeding probiotics containing *Pediococcus acidilactici* (*P. acidilactici*) could modulate bacterial communities related to intestinal health of weaned piglets [11, 12]. However, the combined effect of *L. fermentum* and *P. acidilactici* on weaned pigs has not been investigated extensively. Therefore, we aimed to investigate the combined effects of *L. fermentum* and *P. acidilactici* on growth performance, immune function, short chain fatty acid concentrations, and intestinal bacterial communities in weaned pigs in this study.

## Results

### Growth performance

The probiotics group showed significantly high ADG compared to control and antibiotic groups (*P* < 0.05) over the entire 28 days (Table 1). During the first 14 days, no significant changes in ADFI and F/G were detected among the treatment groups. During day 15–28, the probiotics group consumed less feed and consequently had lower F/G than the other two groups (*P* < 0.05). A lower F/G trend was observed during the entire 28 days in the probiotics group.

**Table 1:** Effect of combined probiotics on the growth performance of weaned pigs^1^

Items	Control	Antibiotic	Probiotics	*P*-value
Initial weight (kg)	7.11 ± 0.86	7.10 ± 0.99	7.13 ± 0.89	0.99
Final weight (kg)	15.25 ± 1.01	15.57 ± 1.34	17.39 ± 1.36	0.03
d1–14				
ADG (g/d)	197.88 ± 4.59^b^	199.47 ± 6.87^b^	247.38 ± 23.47^a^	0.04
ADFI (g/d)	400.33 ± 23.93	376.28 ± 14.84	409.38 ± 21.53	0.45
F/G	2.03 ± 0.14	1.89 ± 0.09	1.70 ± 0.17	0.27
d14–28				
ADG (g/d)	383.75 ± 18.45^b^	405.73 ± 14.87^b^	485.75 ± 23.78^a^	0.01
ADFI (g/d)	739.30 ± 45.70	779.07 ± 30.82	876.33 ± 52.75	0.12
F/G	1.92 ± 0.03^a^	1.92 ± 0.03^a^	1.80 ± 0.04^b^	0.04
d1–28				
ADG (g/d)	290.83 ± 9.47^b^	302.60 ± 9.13^b^	366.58 ± 12.80^a^	< 0.01
ADFI (g/d)	569.80 ± 34.61	577.67 ± 15.42	642.83 ± 36.90	0.19
F/G	1.96 ± 0.06	1.91 ± 0.03	1.75 ± 0.08	0.07
Diarrhea rate^2^	18.10 ± 3.08	19.94 ± 1.79	14.24 ± 6.07	0.16

^1^ *n* = 6 per treatment. All values are means ± SEM

^2^ Diarrhea rate was for overall period

^a, b^ Different letters means there were statistically significant differences among three treatments when *P*-value < 0.05

### Immune function

We observed significant effects of probiotics on levels of inflammatory cytokines in the serum (Table 2). Serum levels of IL-6 (*P* < 0.05) and IFN-γ (*P* < 0.01) in probiotics and antibiotic groups were decreased than the control group. Feeding probiotics exhibited additional effects in down-regulating interleukin-1β (IL-1β) (*P* < 0.05), whereas feeding antibiotic uniquely reduced the serum level of interleukin-10 (IL-10) (*P* < 0.05). Probiotics and antibiotic did not affect serum concentrations of IgA, IgG, or tumor necrosis factor *α* (TNF-α) 28 days after weaning.

**Table 2:** Effect of combined probiotics on the immune response in serum of weaned pigs^1^

Items	Control	Antibiotic	Probiotics	*P*-value
IgA (g/L)	0.89 ± 0.11	0.81 ± 0.07	0.85 ± 0.05	0.34
IgG (g/L)	7.19 ± 1.90	7.1 ± 0.87	7.3 ± 1.34	0.98
IL-10 (pg/mL)	60.60 ± 6.29^a^	46.91 ± 8.42^b^	62.96 ± 1.21^a^	0.02
IL-1β (pg/mL)	48.18 ± 6.29^a^	40.89 ± 5.67^b^	38.45 ± 3.28^b^	0.02
IL-6 (pg/mL)	92.04 ± 8.74^a^	77.89 ± 6.61^b^	72.99 ± 9.97^b^	0.02
TNF-a (pg/mL)	81.55 ± 7.29	75.80 ± 3.89	75.76 ± 2.03	0.17
IFN-γ (pg/mL)	64.50 ± 8.05^a^	48.45 ± 3.83^b^	32.46 ± 8.01^c^	< 0.01

^1^ Serum samples were obtained from one pig randomly selected from each replicate, which means six pigs per group. All values are means ± SEM

^a, b, c^ Different letters means there were statistically significant differences among three treatments when *P*-value < 0.05

### Sequence depth and bacterial diversity of pig intestinal microbiota

A total of 653,050 sequences were generated from 18 digesta samples (9 caecal digesta samples and 9 colonic digesta samples) after noise sequences were discarded according to the minimum sequencing depth. At the 97% sequence similarity, 651 operational taxonomic units (OTUs) were clustered and then allotted to 13 phyla, 21 classes, 34 orders, 57 families, 177 genera and 314 species.

The differences in intestinal bacterial diversity and richness among three groups are shown in Table 3. The probiotics group tended to decrease the caecal bacterial richness (Ace, Chao) and colonic Sobs index without affecting bacterial diversity compared to control group and antibiotic group. In addition, probiotics group had a decreased trend in bacterial community richness (Sobs) and the bacterial diversity (Shannon, Simpson) of the colonic digesta. The bacterial diversity and richness of antibiotic group fell in between the control group and probiotics group.

**Table 3:** Alpha-diversity of bacterial community in the caecal and colonic digesta of weaned pigs^1^

Sample	Control	Antibiotic	Probiotics	*P-*value
Caecal digesta				
Sobs	420.00 ± 24.88	364.00 ± 39.15	315.00 ± 58.80	0.08
Ace	484.93 ± 28.01	416.52 ± 44.75	366.36 ± 58.34	0.05
Chao	484.75 ± 30.28	420.03 ± 36.42	373.23 ± 68.57	0.05
Shannon	4.40 ± 0.24	3.77 ± 0.58	3.71 ± 0.60	0.18
Simpson	0.03 ± 0.01	0.07 ± 0.06	0.07 ± 0.04	0.43
Colonic digesta				
Sobs	475.33 ± 9.07	427.33 ± 47.06	381.67 ± 34.65	0.05
Ace	521.66 ± 23.2	476.79 ± 43.89	446.49 ± 34.49	0.11
Chao	529.69 ± 30.55	480.54 ± 50.12	476.30 ± 60.38	0.30
Shannon	4.62 ± 0.07	4.08 ± 0.38	3.74 ± 0.50	0.06
Simpson	0.03 ± 0.002	0.05 ± 0.03	0.09 ± 0.06	0.06

^1^ Caecal and colonic digesta samples were obtained from three pigs per group and their microbiota composition were analyzed using 16S rRNA sequencing. All values are means ± SEM

**Fig. 1:**
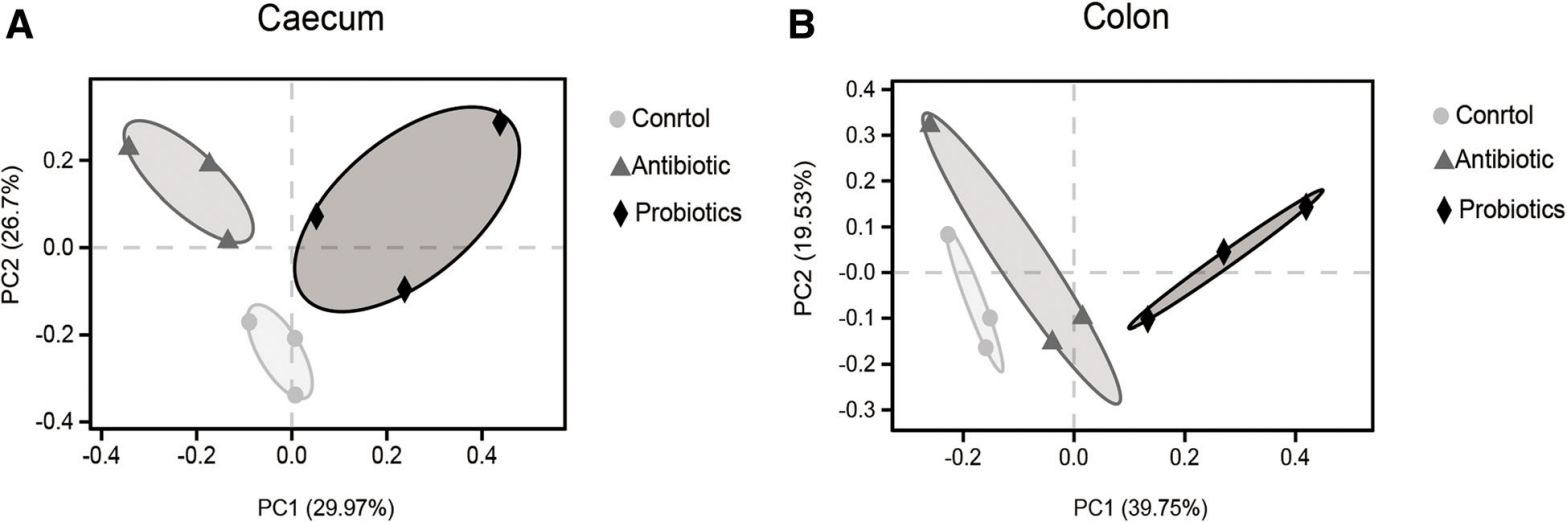
Principal coordinate analysis (PCoA) based on Bray_curtis distance. Different symbols represent different groups. (**a**) PCoA plot for the caecal digesta microbial communities. (**b**) PCoA plot for the colonic digesta microbial communities. Circles: control group; triangles: antibiotic group; rhombus: probiotics group

For beta-diversity analysis, PCoA based on Bray-Curtis distances was performed in caecal and colonic microbiota collected from pigs of the three groups. The result (Fig. 1) showed that the microbiota from pigs of probiotics group was separated from those in the control and antibiotic groups.

### Core bacteria of weaned pigs

**Fig. 2:**
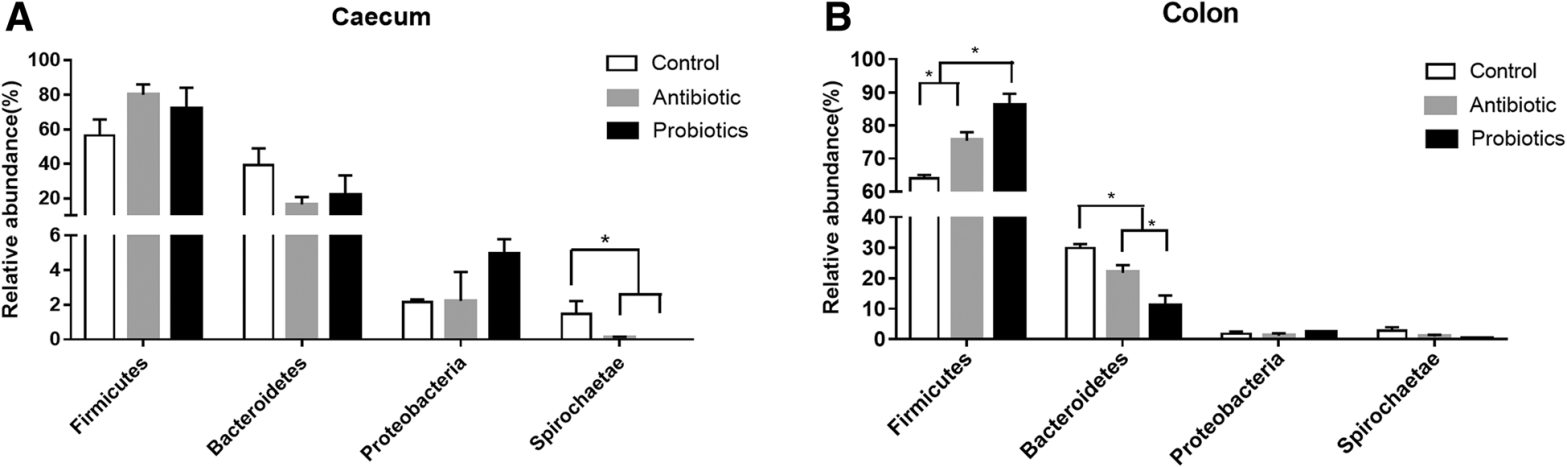
Dominant phylum of caecal and colonic digesta obtained from weaned pigs (**a**) Changes in caecal microbiota composition of control, antibiotic, probiotics group at the phylum level. (**b**) Changes in colonic microbiota composition of control, antibiotic, probiotics group at the phylum level. “*” means there were statically significant differences (*P* < 0.05)

**Fig. 3:**
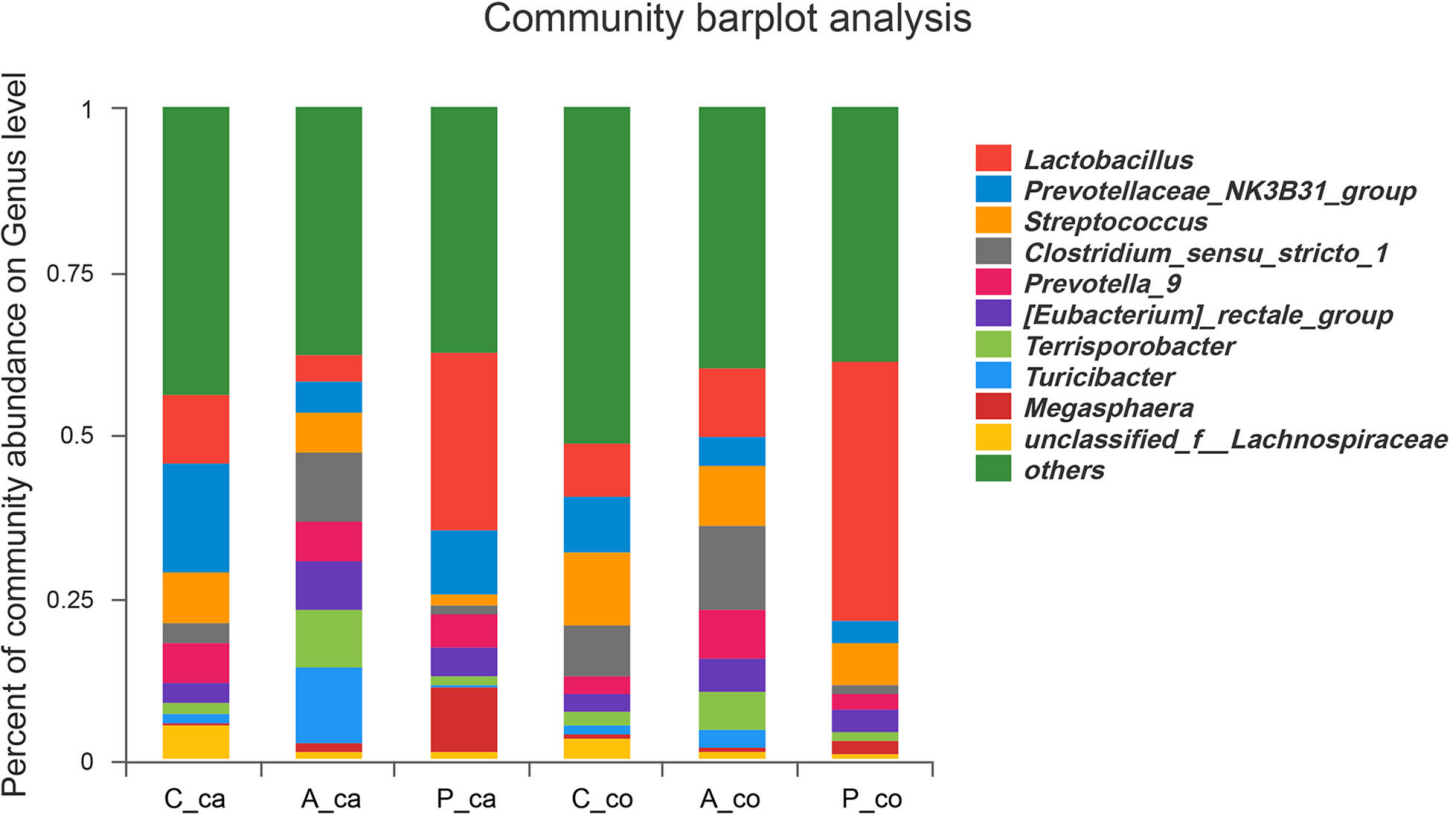
Characterization of communities on genus level. Caecal and colonic relative abundance of microbial genus of weaned piglets fed the basic diet (control), antibiotics and probiotics. (C_ca: caecal samples of control group; A_ca: caecal samples of antibiotic group; P_ca: caecal samples of probitocs group; C_co: colonic samples of control group; A_co: colonic samples of antibiotic group; P_co: colonic samples of probitocs group)

At the phylum level, Firmicutes, Bacteroidetes, and Proteobacteria were dominating in caecal and colonic digesta in all three groups, which included 99% of taxa. Spirochaetae, rich in the control group, was nearly undetectable in the probiotics and antibiotic groups (Fig. 2). At genus level, *Lactobacillus* (27.30%) was more abundant in the caecal digesta of piglets fed combined *L. fermentum* and *P. acidilactici* than that in control (10.48%) and antibiotic groups (4.07%). Similarly, in the colonic digesta, *Lactobacillus* was more abundant in probiotics group (39.90%) than in the control (8.36%) and antibiotic groups (10.51%) (Fig. 3). *Prevotellaceae_NK3B31_group*, *Lactobacillus* and *Streptococcus* were the most abundant three genera in both caecal and colonic digesta in the control group, while *Clostridium_sensu_stricto_1* was common dominant of the antibiotic group in both caecal and colonic digesta.

### Changes in microbial composition after probiotics administration

**Fig. 4:**
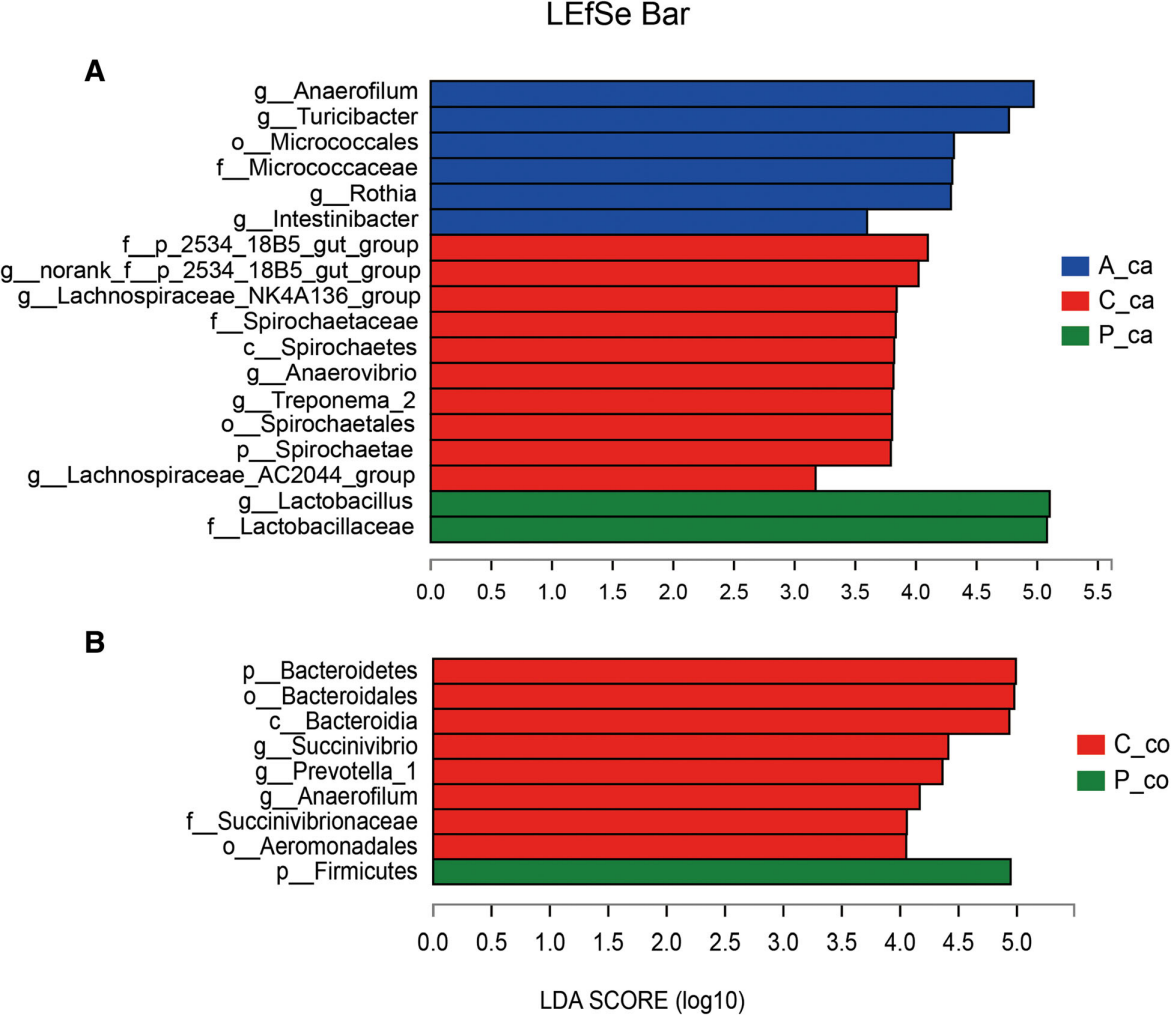
Linear discriminant analysis effect size (LEfSe) analysis microbiota in the caecal and colonic chyme of weaned pigs from phylum to genus levels. The linear discriminant analysis (LDA) plots indicate species that can be used as biomarkers, which was determined by ranking them according to their effect size. Different colors represent different groups. (**a**) LDA analysis of the caecum microbial. C_ca (caecal samples of control group), red bars; A_ca (caecal samples of antibiotic group), blue bars; P_ca (caecal samples of probiotics group), green bars. (**b**) LDA analysis of the colonic microbial. C_co (colonic samples of control group), red bars; P_co (colonic samples of probiotics group), green bars

To clearly identify how the bacteria changed among the three treatments, the linear discriminant analysis (LDA) effect size (LEfSe) were used to evaluate the differences in the relative abundance from phylum to genus (Fig. 4).

In the caecal digesta, proportion from the Lactobacillaceae family to *Lactobacillus* genus was significantly increased by combined *L. fermentum* and *P. acidilactici* supplementation, while greater relative abundance of *Treponema_2* from Spirochaetae phylum, *Anaerovibrio* genus and two strains from *Lachnospiraceae* were observed in the control group. Besides, the abundance of *Anaerofilum*, *Turicibacter*, *Rothia* and *Intestinibacter* genus were increased significantly in the antibiotic group.

In the colon, the abundance of Firmicutes phylum was higher in probiotics group compared to the control group, while the relative abundance of *Anaerofilum*, *Prevotella_1* from Bacteroidetes phylum and *Succinivibrio* from Aeromonadales order were lower.

### SCFAs concentrations

The concentrations of 4 short chain fatty acids in the caecal and colonic digesta of pigs were measured (Table 4). Compared with the other two groups, acetic acid and propionic acid in caecal digesta of probiotics fed pig were higher (*P* < 0.05). No significant differences in caecal lactic acid and butyric acid were detected among the three groups. As for the concentrations of SCFAs in colonic contents, contents of lactic acid and propionic acid of the antibiotic group were significantly lower than the other two groups (*P* < 0.05). There was no statistical difference in the colonic acetic acid and butyric acid among the three groups.

**Table 4:** Concentrations of short chain fatty acids in the caecal and colonic digesta of weaned pigs^1^

Item	Control	Antibiotic	Probiotics	*P-*value
Caecal digesta				
lactic acid (mg/g)	2.66 ± 0.52	2.57 ± 0.63	2.91 ± 0.03	0.67
Acetic acid (mg/g)	5.29 ± 0.58^b^	5.32 ± 0.28^b^	6.23 ± 0.49^a^	0.03
Propionic acid (mg/g)	3.02 ± 0.70^b^	2.92 ± 0.24^b^	3.73 ± 0.45^a^	0.02
Butyric acid (mg/g)	1.32 ± 0.39	1.75 ± 0.48	1.54 ± 0.41	0.34
Colonic digesta				
lactic acid (mg/g)	5.30 ± 0.54^a^	3.87 ± 0.66^b^	5.60 ± 0.53^a^	0.01
Acetic acid (mg/g)	5.93 ± 0.33	5.92 ± 0.48	6.49 ± 0.59	0.21
Propionic acid (mg/g)	3.02 ± 0.41^a^	2.53 ± 0.30^b^	3.07 ± 0.30^a^	0.04
Butyric acid (mg/g)	1.54 ± 0.22	1.76 ± 0.28	1.60 ± 0.25	0.47

^1^ The digesta samples were obtained from the caecum and colon of three pigs per group and the concentrations of SCFAs were measured. All values are means ± SEM

^a, b^ Different letters means there were statistically significant differences among three treatments when *P*-value < 0.05

## Discussion

Probiotics, when administered in proper proportion, can modulate the intestinal microbial population, reduce diarrhea and boost the immune system of the host, which can then enhance gut health and growth performance of pigs [13, 14]. There have been some previous studies focusing on identifying the beneficial effect of individual probiotic specie *L. fermentum and P. acidilactici* fed individually as probiotics for animals [8, 9, 11, 12]. However, whether the combined *L. fermentum* and *P. acidilactici* could complement individual effects to each other and modulate gut bacterial community better have not been studied. Therefore, we tried to understand the effects of dietary supplementation of combined *L. fermentum* and *P. acidilactici* preparation on weaned pigs in the present study. Weaned piglets were fed a basal diet, or supplemented with chlortetracycline or combined *L. fermentum* and *P. acidilactici* in the current study. Our results showed that supplementing diets with probiotics could improve growth performance, regulate inflammation through lowering pro-inflammatory factors, and change the composition of intestinal microbiota.

Weaning may be accompanied with diarrhea and growth inhibition in piglets [2], growth performance is one of the most important aspects of animal performance in the pig industry. In this study, we observed that weaned pigs supplemented with combined *L. fermentum* and *P. acidilactici* preparation demonstrated greater ADG for the whole 28 days and improved feed efficiency in the latter half of the trial compared with weaned pigs in the other two groups. A previous study identified that administration of *Pediococcus acidilactici FT28* enhanced feed conversion rate of weaned piglets compared with the control group [15], which corresponds with our result but it had no significant effect on the ADG. Our finding is also supported by earlier research that weaned pigs supplemented with probiotic complexes containing *Enterococcus faecium, Bacillus subtilis* and *Lactobacillus paracasei* had greater ADG and improved feed efficiency [16]. Therefore, it can be speculated that combined probiotics exhibited additional benefits on growth performance compared with a single probiotic specie. Besides, *L. fermentum* I5007 was identified in enhancing the levels of proteins in relation to energy metabolism and protein synthesis of weaned piglets, whereas chlortetracycline reduced them [10], which may account for the different growth performance between the probiotics group and the antibiotic group.

Growth performance of animals is also relevant to their health status and immunity. Both probiotics group and antibiotic group were followed by the reduction in the serum concentrations of IL-6 and IFN-γ compared with control group. Probiotics group uniquely decreased IL-1β compared with the other two groups. The pro-inflammatory cytokines IL-1β and IL-6 are significant in the immune response and the tissue maintenance [17–19]. In agreement with this study, it was reported that *Lactobacillus reuteri* and *L. fermentum* could depress the expression of pro-inflammatory factor TNF-α in a rat colitis model [20]. In another study, the control group not receiving probiotics had an increased expression of pro-inflammatory cytokines [21]. Interestingly, the anti-inflammatory cytokine, IL-10 in serum, was reduced in the antibiotic group. The IL-10 is an important immune regulator in the gut tract, which is mainly released by B lymphocytes [22]. All in all, probiotics helped to regulate the inflammatory response, reduce inflammatory damage and then improve health of weaned pigs, which performed better than antibiotic fed pigs.

When it comes to the intestinal microbiota, we mainly focused on the microbiota from caecal and colonic digesta of the weaned pigs where microbial population is abundant and diverse [13]. Our results showed that combined *L. fermentum* and *P. acidilactici* tended to decrease the alpha diversity of intestinal microbiota in a similar trend with antibiotic, which may be due to the fact that probiotics can inhibit pathogens and modulate intestinal microbiota.

Firmicutes, Bacteroidetes, Proteobacteria and Spirochaetae were the top four phyla in both caecal and colonic digesta. These results were consistent with a previous study [23], where Firmicutes and Bacteroidetes were most dominant. In the caecal digesta, the relative abundance of *Treponema_2* genus and its phylum Spirochaetae were significantly abundant in the control group than the probiotics and antibiotic groups. Spirochaetae was nearly undetectable in the other two groups, similar to a previous study in which pigs were fed *Lactobacillus salivarius* UCC118 WT [24]. One genus of the spirochetes was identified to be related to swine diarrhea [25]. Thus the ability of the combined *L. fermentum* and *P. acidilactici* to control destructive bacteria could be significant, which may benefit growth. In colonic digesta, we found that both the probiotics group and antibiotic group significantly increased the abundance of Firmicutes and decreased the abundance of Bacteroidetes. These results were consistent with a previous study which showed that establishment of Firmicutes in ileal microbiota of weaned pigs was promoted by *P. acidilactici* and antibiotic treatment [11]. The phyla Firmicutes and Bacteroidetes are known for polysaccharide fermentation. When intestinal Firmicutes / Bacteroidetes ratio was improved, the host are able to absorb more energy from the diet and the ability to store energy are strengthened [26, 27]. The existence of the common greater beneficial phyla and the less potential pathogen in the probiotics group may be potential factors involved in the increased body weight gain in the probiotics group.

*Lactobacillus*, *Prevotellaceae_NK3B31_group*, *Streptococcus* and *Clostridium_sensu_stricto_1* were the dominant genera in three groups, but the most dominant genera were different in each group. Lactobacillaceae family and *Lactobacillus* genus did affect the caecal microbiota of the probiotics group. *Lactobacillus* can produce energy through glycolysis and ferment carbohydrates into lactic acid without oxygen [28]. Although there was no statistical difference in lactic acid concentration of caecal digesta, it may contribute to the higher SCFAs (acetic acid and propionic acid) levels in the caecal digesta. Similar results were showed in a previous study about probiotic complexes containing *Enterococcus faecium, Bacillus subtilis* and *Lactobacillus paracasei* [16]. Meanwhile, the *Lactobacillus* level tended to improve in the colonic digesta, which partly explained higher lactic acid and propionic acid than the antibiotic group. Some strains of *Lactobacillus* have been found possess anti-bacterial activities, anti-inflammation [29], and increased *Lactobacillus* in the intestinal digesta [30]. The *Lactobacillus* promoted the production of SCFAs, suppressed pathogens with an acidic environment and digested nutrients that the host cannot metabolize, which ultimately improves intestinal health status and growth.

In addition, 5 genera in the caecal digesta and 3 genera in the colonic digesta were more abundant in the control group compared to the other two groups. Of these unique genera, A*naerovibrio*, an anaerobic bacteria belonging to the Negativicutes class, was higher in the control group. The members of Negativicutes have a peculiar cell wall composition which is Gram negative [31]. Interestingly, lactic acid produced by *Lactobacillus* is vital to antimicrobial activities for suppressing the generation and growth of Gram-negative bacteria virulence factors [32, 33]. The combined *L. fermentum* and *P. acidilactici* may decrease the *Anaerovibrio* by promoting lactic acid production, which permeated the gram-negative bacterial outer membrane [34]. The reduction of pathogenic microbial load in intestines could limit the negative influence of microbiota towards its host, which may improve growth performance of pigs [35]. In the colonic digesta, the high abundance *Prevotella_1 and Succinivibrio* genus in control group are the producer of acetic acids and succinic acids, which were end-products of fermentation [36]. *Succinivibrio* was sensitive to antibiotics [37], which partly explains the lower colonic lactic acid and propionic acid in the antibiotic group.

In the antibiotic group, caecal bacterial abundance of 4 genera increased compared to the other two groups. For example, the abundance of *Rothia* genus and its order Micrococcales were higher. *Rothia spp.* cause extensive severe infections, particular in immunocompromised hosts [38], which may be related to lower serum IL-10 level. All in all, the antibiotic modulated the intestinal microbiota by inhibiting specific bacteria, but there were still potentially harmful bacteria, owning to its disturbance of the normal balance of gut microflora. Therefore, the growth performance of antibiotic group was not as good as that of the probiotics group, which also provided information that the combined *L. fermentum* and *P. acidilactici* has potential preventive effect like antibiotic and could improve pig performance.

A potential limitation of this study is that the sample size of sacrificed pigs for the SCFAs concentrations and gut microbiome analysis is a little small, but some tests indicated a difference among three groups. We will carry out a larger sample study in the future and focus on the SCFAs concentrations and gut microbiome analysis test.

## Conclusions

In conclusion, dietary supplementation with combined *L. fermentum* and *P. acidilactici* improved ADG and F/G of weaned pigs. Concentrations of the serum pro-inflammatory factors IL-6, IFN-γ were significantly decreased in the probiotics group and antibiotic group compared with the control group. In addition, the probiotics group enriched the abundance of *Lactobacillus* in the caecal digesta and Firmicutes in the colonic digesta, which may be related to greater concentrations of caecal SCFAs in the probiotics group. In addition, the growth of *Treponema_2*, *Anaerovibrio* in the caecal digesta was inhibited in the probiotics and antibiotic groups. These findings suggest that combined *Lactobacillus fermentum* and *Pediococcus acidilactici* supplementation of diets improved growth performance, alleviated inflammation and regulated gut health by promoting beneficial bacteria, inhibiting pathogens, and promoting the production of SCFAs in weaned pigs.

## Methods

### Animals, diets and experimental design

Animal protocols were conducted on the basis of the regulations of China Agricultural University Animal Care and Use Committee (Beijing, China). The weaned pigs were owned by FengNing Swine Research Unit of China Agricultural University (Academician Workstation in Chengdejiuyun Agricultural and Livestock Co., Ltd).

One hundred and eight pigs (Duroc × Landrace × Large White) were weaned at 28 d of age (7.12 ± 0.08 kg), were divided into three groups with randomized design considering the litter of origin and gender. Piglets from the same litter were assigned to different treatment group. There were 6 replicate pens per treatment group and 6 pigs per pen (half male and half female). There was no statistical difference among the average initial weight of weaned pigs in each group (*P* = 0.99). The animal size was based on a published study [39]. The experimental treatments were as follows (Additional file 1: Table S1): i. Control: basal diet; ii. Antibiotic: the basal diet plus 75 mg· kg^− 1^ chlortetracycline (commercially available chlortetracycline with a purity of 15%); and iii. Probiotics: basal diet plus 4% compound probiotics. The different diets were started to be supplied to the pigs weaned at 28 d of age and the experimental period lasting for 28 days. Pigs were fed in a nursery house with plastic leakage dung floors. Water and feed were supplied ad libitum during the experimental period.

### Probiotics compound preparation

The *L. fermentum* and *P. acidilactici*-containing probiotics compound preparation was provided by Hebei Daguang Biotechnology Co. LTD, which is a fermented feed additive. The combined probiotics preparation were cultured and fermented into liquid, then the fermentation substrates including 60% wheat bran, 10% corn meal, and 30% soybean meal were added and the solid-liquid combinations were fermented. The combined probiotics preparation was stored in the feed fermentation bag with single exhaust valve for maintaining its natural high activity. The final counted viable bacteria was 1.6 × 10^9^ CFU/g, mainly including 9.1 × 10^8^ CFU/g *Lactobacillus fermentum* and 5.25 × 10^8^ CFU/g *Pediococcus acidilactici*. The pigs were fed four times per day at 8:30, 11:30, 14:30 and 17:30. The compound probiotics preparation was mixed with feed in a proportion of 4% just before feeding the pigs.

### Sample collection

All pigs were weaned at the 28 d of age and the diet treatment started from weaning day and lasted for 28 days. During the experiment period, pig were weighed individually at d 1, d 14 and d 28 after weaning. Feed consumption of each pen was monitored. Average daily gain (ADG), average daily feed intake (ADFI) and feed consumed/weight gain (F/G) during d 1 to d 14 and d 14 to d 28, were calculated to observe the growth performance of piglets. And the diarrhea rate was calculated based on the fecal score system as follows: 0, normal consistency; 1, pasty; 2, semiliquid; and 3 liquid. Pigs with a score of 2 or greater were considered to have diarrhea [40]. Diarrhea rate (%) = 100 * [total number of diarrhea cases in each pen during the trial period / (total number of pigs in each pen * total number of days in the trial)].

On the d 28, one pig was selected randomly from each replicate pen to collect blood samples from the jugular vein. After blood samples were naturally coagulated, they were immediately centrifuged at 3000 g for 10 min, and then removed and placed in − 20 °C freezer to be analyzed for immunoglobulins and inflammatory cytokines.

Three pigs per treatment were randomly selected. The selected pigs were from the different pens and their body weights were representative of the average body weights. The sample size for intestinal microbiota analysis was based on a related study [3]. The pigs were sacrificed after exsanguination under anesthesia by intravenous injection with sodium pentobarbital (40 mg/kg BW) in a slaughter house. Approximately 10 g digesta samples were collected into sterile tubes from the mid colon and caecum of each pig. Digesta samples were stored in liquid nitrogen immediately before being removed to the laboratory and stored at − 80 °C freezer until the analysis of short chain fatty acids (SCFAs) concentrations and microbiota composition. The investigators were blinded to group allocation during the experiments analysis.

### Analysis of immunoglobulins and inflammatory cytokines of serum samples

Serum concentrations of IgA (144118001; Mindray Co., Ltd., Shenzhen; China) and IgG (14438002; Mindray Co., Ltd., Shenzhen; China) on day 28 were analyzed by a Hitachi 7600 automatic biochemical analyzer. The levels of interleukins interleukin-1β (IL-1β), interleukin-10 (IL-10) and interleukin-6 (IL-6), interferon-γ (IFN-γ), and tumor necrosis factor 훼 (TNF-α) were measured using commercial ELISA kits for pigs following the manufacturer’s instructions (Konka Hongyuan Biotechnology Co., Ltd., Beijing; China).

### Microbial analysis

#### DNA extraction and PCR amplification and sequencing

Microbial DNA was extracted from digesta using the QIAamp R Fast DNA Stool Mini Kit (Qiagen Ltd., Germany) according to manufacturer’s instructions. The final DNA quality were examined by 1% agarose gel electrophoresis and NanoDrop 2000 UV-vis spectrophotometer (Thermo Scientific, Wilmington, USA). The V3-V4 regions of the bacteria 16S rRNA gene were generated with universal primers 338F (5′-ACTCCTACGGGAGGCAGCAG-3′) and 806R (5′-GGACTACHVGGGTWTCTAAT-3′) by PCR amplification [41]. The PCR reactions were conducted as follows: denaturation at 95 °C for 3 min, 27 cycles at 95 °C for 30 s, 30 s for annealing at 55 °C, and elongation at 72 °C for 45 s, and a final extension at 72 °C for 10 min. Purified amplicons were pooled in equimolar and paired-end sequenced on the Illumina MiSeq platform (Illumina, San Diego, USA) [42]. All the raw data were uploaded into NCBI Sequence Read Archive database with accession number PRJNA495019. The raw sequencing data were processing in accordance with a previous study [41].

OTUs were clustered with a 97% similarity cutoff using UPARSE(version 7.1 http://drive5.com/uparse/) and chimeric sequences were identified and removed using UCHIME. The taxonomy of each 16S rRNA gene sequence was analyzed by RDP Classifier algorithm (http://rdp.cme.msu.edu/) against the Silva 128/16s_bacterial database (http://www.arb-silva.de) using confidence threshold of 70%. The alpha-diversity including richness, diversity and coverage based on the Sobs, Chao, Ace, Shannon, Simpson and Coverage index within each sample were generated by mothur (version v.1.30.1). Principal coordinate analysis (PCoA) were calculated based on the Bray-Curtis distances.

### Digesta SCFAs analysis

The concentrations of SCFAs in the digesta sample of each pig were analyzed in the high-performance ion chromatography system (DIONEX ICS-3000; Thermo Fisher, Waltham, MA; USA) [43]. Approximately 0.5 g of digesta content was weighed into a 10 ml centrifuge tube. Each sample was weighed for two tubes. Ultrapure water (8 ml) was added and then vortexed to mix the contents. After centrifugation at 4000 r/min for 15 min, 0.16 ml supernatant was removed into a 10 ml centrifuge tube, and 7.84 ml of ultrapure water solution (equivalent to a 50-fold dilution) was added. Then filtered the dilution with a 0.22 μm membrane and analyzed (Dionex IonPac AS11-HC; Thermo Fisher, Waltham, MA; USA). The concentrations of short chain fatty acids were calculated.

### Statistical analysis

The data of growth performance, inflammatory cytokines, and SCFAs were subjected to an analysis of variance method using SAS software (Windows V8) with general linear model procedure, comparing the differences among three groups. Six pigs in one pen were considered as the experimental unit of analyses for the difference in growth performance. As for inflammatory cytokines, SCFAs and microbial analysis, individual pigs were considered as the experimental unit. Outliers were eliminated based on 3δ criterion. Significant differences among means of groups were determined by Duncan’s multiple range tests. *P*-values < 0.05 were considered as statistically significant. All values are presented as means ± SEM. The difference in the alpha diversity among three groups was tested using Kruskal-Wallis (KW) test (SAS Windows V8) and *P*-values were adjusted with FDR when they were < 5%. LEfse was used to conduct linear discriminant analysis (LDA) to estimate the effect of abundance of each component (species) on the differences. And the multi-group comparison strategy was all-against-all.

## Additional file

Additional file 1: **Table S1.** Diet composition and nutrient levels^1^. (DOCX 16 kb)

## Data Availability

The 16S rRNA gene sequences raw datasets generated during the current study are available in the NCBI Sequence Read Archive (SRA) database with accession number PRJNA495019. The diet composition and nutrient levels are included within its supplementary information files. The other datasets used during the current study are available from the corresponding author on reasonable request.

## Abbreviations

ADFI: Average daily feed intake
ADG: Average daily gain
F/G: Feed consumed/weight gain
IFN-γ: Interferon-γ
IL-10: Interleukin-10
IL-1β: Interleukin-1β
IL-6: Interleukin-6
LDA: Linear discriminant analysis
LEfSe: Linear discriminant analysis effect size
OTUs: Operational taxonomic units
PCoA: Principal coordinate analysis
SCFAs: Short chain fatty acids
TNF-α: Tumor necrosis factor-α
