# Supplementary material for: Combined supplementation of Lactobacillus fermentum and Pediococcus acidilactici promoted growth performance, alleviated inflammation, and modulated intestinal microbiota in weaned pigs
Source: BMC Vet Res. 2019 Jul 10;15:239. doi: 10.1186/s12917-019-1991-9 (PMC6617942; doi:10.1186/s12917-019-1991-9)
Supplement: Supplementary file 1 — Table S1. Diet composition and nutrient levels1. (DOCX 16 kb) [file 12917_2019_1991_MOESM1_ESM.docx]

# Additional file 1

# Table S1. Diet composition and nutrient levels ^1^

| Items | Control | Antibiotics | Probiotics |
| --- | --- | --- | --- |
| Feedstuffs (%) |  |  |  |
| Corn | 58.95 | 58.90 | 54.15 |
| Soybean meal | 11.00 | 11.00 | 11.80 |
| Extruded full - fat soybean | 8.00 | 8.00 | 9.00 |
| Fish meal | 2.00 | 2.00 | 0.00 |
| Dried whey | 6.00 | 6.00 | 6.00 |
| Soybean protein concentrate | 5.00 | 5.00 | 6.00 |
| Soybean oil | 0.50 | 0.50 | 0.50 |
| Premix^2^ | 4.00 | 4.00 | 4.00 |
| Glucose | 4.00 | 4.00 | 4.00 |
| Cr_2_O_3_ | 0.25 | 0.25 | 0.25 |
| Lys | 0.10 | 0.10 | 0.10 |
| Met | 0.10 | 0.10 | 0.10 |
| Thr | 0.10 | 0.10 | 0.10 |
| Probiotics^3^ |  |  | 4.00 |
| Antibiotic^4^ |  | 0.05 |  |
| Total | 100.00 | 100.00 | 100.00 |
| Calculated analysis^5^ |  |  |  |
| Crude protein (%) | 17.39 | 18.76 | 17.86 |
| ME( MJ/kg) | 16.55 | 16.53 | 16.37 |
| Ca (%) | 0.83 | 0.83 | 0.82 |
| P (%) | 0.66 | 0.65 | 0.66 |
| Lys (%) | 1.42 | 1.42 | 1.42 |
| Met (%) | 0.40 | 0.40 | 0.40 |

^1^ Without adding antibiotics in the feed ingredients

^2^ The premix vitamin and mineral content in per kg diet is as follows: vitamin A, 5,000 IU; vitamin D3, 1,350 IU; vitamin E, 13.5 IU; vitamin K3, 1.45 mg; vitamin B12, 9 μg; riboflavin, 2.7 mg; pantothenic acid, 8.0 mg; niacin, 16 mg; choline chloride, 280 mg; folacin, 0.3 mg; thiamine 0.7 mg; pyridoxine 1.35 mg; biotin, 20 μg; Mn, 15.0 mg (MnO); Fe, 70 mg (FeSO4•H2O); Zn, 65 mg (ZnO); Cu, 25 mg (CuSO4•5H2O); I, 0.3 mg (KI); Se, 0.2 mg (Na2SeO3) [[43](#_ENREF_43)].

^3^ Probiotics: *Lactobacillus fermentum*: *Pediococcus acidilactici* =2:1. The viable cell count of the bacteria was 1.6 × 10^9^ CFU/g. The combined *L. fermentum* and *P. acidilactici* preparation were cultured and fermented into liquid, and then solid-liquid combination was fermented with bran, soybean meal and corn flour.

^4^ Antibiotic: 75 mg/kg chlortetracycline (commercially available chlortetracycline with a purity of 15%);

^5^ The nutrients levels were calculated values.
